# Choroid Plexus Papilloma Mimicking Chiari II Malformation in an Infant With Myelomeningocele: A Case Report

**DOI:** 10.1155/crpe/4584232

**Published:** 2026-11-13

**Authors:** Harris Shahid, Priya Kadam, Chandrasekaran Kaliaperumal

**Affiliations:** ^1^ Edinburgh Medical School University of Edinburgh 49 Little France Crescent Edinburgh EH16 4SB UK ed.ac.uk; ^2^ Department of Clinical Neurosciences Royal Infirmary of Edinburgh 50 Little France Crescent Edinburgh EH16 4SA UK nhs.uk; ^3^ Royal Hospital for Children and Young People 50 Little France Crescent Edinburgh EH16 4SA UK

**Keywords:** central sleep apnoea, Chiari II malformation, choroid plexus papilloma, myelomeningocele, spina bifida

## Abstract

We describe a rare case of an infant with a diagnostic conundrum of apnoeic episodes, on the background of antenatally diagnosed myelomeningocele and Chiari II malformation (CM2). The infant underwent extensive neurosurgical interventions in the first few weeks of life and treatment including foramen magnum decompression of CM2 and placement of a ventriculoperitoneal shunt for hydrocephalus, with an incidental finding of a choroid plexus papilloma (CPP) WHO Grade 1. The apnoeic episodes persisted and required multidisciplinary discussion, considering the relative likelihood of competing aetiologies. Investigations eventually led to the finding that urinary tract infection was exacerbating the apnoeic episodes and may have contributed to the frequency or severity of the apnoeic episodes. From a neurosurgical perspective, we discuss whether CM2, the fourth‐ventricular CPP, and associated hydrocephalus may have contributed to brainstem dysfunction and respiratory dysregulation. From a neurosurgical perspective, we discuss the potential contributions of CM2, a fourth‐ventricular CPP, hydrocephalus, and brainstem dysfunction to the infant’s respiratory events. The temporal association between urinary tract infection and increased apnoeic episodes also raises the possibility of bladder‐mediated autonomic dysregulation, although a single definitive aetiology was not established.

## 1. Introduction

Choroid plexus papilloma (CPP) is a rare benign ventricular tumour, which often leads to the formation of hydrocephalus via cerebrospinal fluid (CSF) overproduction or obstruction. While CPPs in infants typically arise in the lateral ventricles, the presence of a fourth ventricle CPP is uncommon [1], and its coexistence with Chiari II malformation (CM2) is even rarer.

CM2 results from a failure of neural tube closure, leading to hindbrain herniation and associated hydrocephalus, often requiring ventriculoperitoneal shunting (VPS) and foramen magnum decompression. Several complications may arise from CM2, including central sleep apnoea (CSA), which is likely due to brainstem compression involving the respiratory centres (Figure 1). In addition, there are long‐term cognitive impairments which may result from congenital neural tube defects and CM2, including visuospatial skills and slower cognitive processing [2], and early identification and intervention can improve prognosis [2].

**FIGURE 1:**
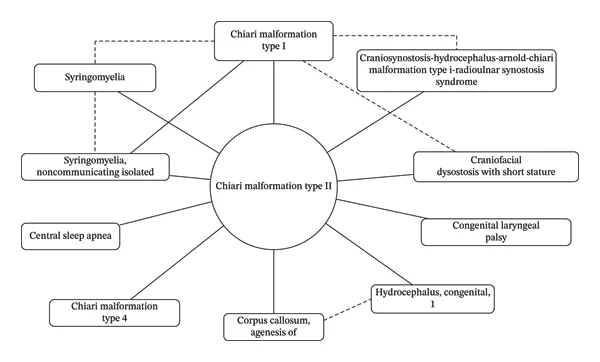
Commonly reported associations and complications of Chiari II malformation, including central sleep apnoe, adaped from [3].

It was found that sleep‐disordered breathing affects more than half of neonates with myelomeningocele; in a nine‐centre prospective observational study with 173 neonates with the myelomeningocele, it was observed that there were more apnoeic events foetal repair compared with postnatal surgery [4].

## 2. Case Study

### 2.1. Birth and Early Surgery

A premature infant born at 33 + 4 weeks with a weight of 2.1 kg and a head circumference of 33.5 cm was antenatally diagnosed with myelomeningocele and CM2 (Figure 2). She was born to healthy parents with no past medical history of note. She deteriorated shortly after birth and required inflation breaths and chest compressions and was subsequently intubated and ventilated.

**FIGURE 2:**
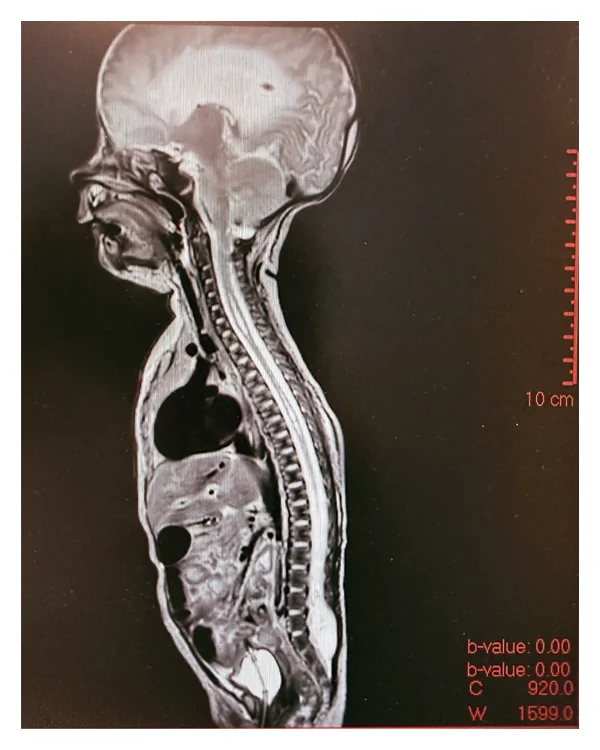
T2‐weighted sagittal MRI scan demonstrating Chiari II malformation and cervical and thoracic syringomyelia.

A CSF leak was noted at spinal level L3/4 measuring 2 × 1.5 cm, and she successfully underwent repair of the myelomeningocele with no complications.

It was observed on the following day that she was experiencing episodes of bradycardia, ‘neopuffing’ and apnoeas two to three times per day, and was placed on intermittent 25% oxygen to maintain her saturations. Looking for the cause of these apnoeas, they appeared unrelated to both feeding and infection. A trial of intravenous Tazocin (piperacillin–tazobactam) and vancomycin was initiated for 2 days and stopped after a negative blood culture, and low CRP was found. No organisms were seen on CSF microscopy, and CSF culture was negative after a 16 mL tap.

See Figure 3 for a timeline of events in this case study.

**FIGURE 3:**
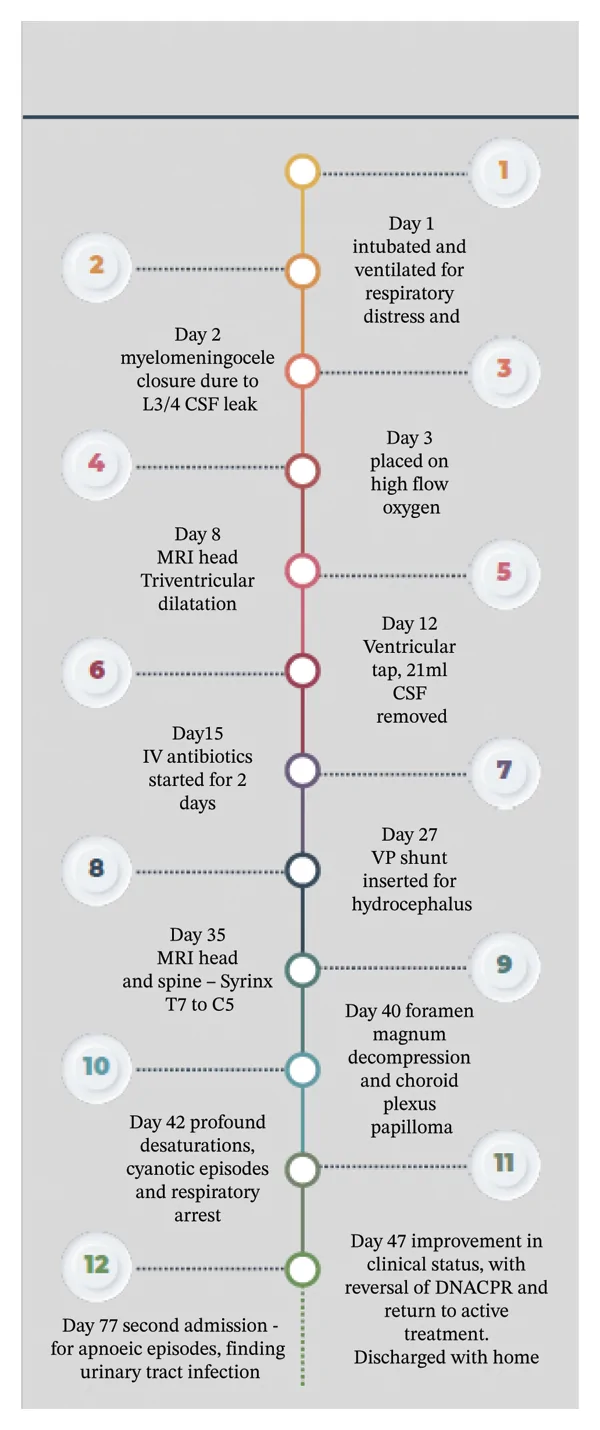
Timeline of events from Day 1 of life to second admission.

### 2.2. Further Investigation of Apnoeic Episodes

The twice‐daily apnoeic episodes persisted and were investigated further, with a normal airway on nasoendoscopy, normal echocardiogram and electroencephalogram. Similarly, a bronchoscopy and microlaryngobronchoscopy revealed no pathology.

The patient was monitored twice weekly from a neurological perspective, with regular cranial ultrasound scans, to establish any changes in neurological function.

### 2.3. Hydrocephalus and VP Shunt Insertion

The MDT continued to search for a cause of the apnoeic episodes. The observation of full body rigidity during apnoeic episodes led to an MRI of the head and spine.

An MRI head was performed on the 8th day of life, which revealed a CM2 with triventricular hydrocephalus. A ventricular tap was performed 4 days later, and 21 mL of CSF was removed. Once more, there was no growth on the CSF culture.

A VPS was inserted on Day 27 due to persistent hydrocephalus, following which incubator oxygen (FiO_2_; 24%–28%) was required before transitioning to a low flow of nasal cannula oxygen at 0.2 L/min. However, multiple episodes of desaturations continued along with bradycardia, normal CRP and blood cultures. Again, she received 36 h of antibiotics, whilst awaiting results of her blood cultures.

### 2.4. Syringomyelia

MRI spine (Figure 2) demonstrated tonsillar descent to at least T4, appearing to merge with the thoracic syrinx ending at T7 from C5 giving the appearance of syringomyelia. Although this was an incidental finding, the CM2 and the syringomyelia may have been associated with one another. No surgery was performed for this pathology at the time.

In addition, a small amount of free fluid in the abdomen from the VP shunt was noted, which was not thought to be related to the apnoeic episodes and at the time did not necessitate intervention.

### 2.5. Foramen Magnum Decompression and Incidental Finding of Tumour

After multidisciplinary team discussion, she underwent a foramen magnum decompression for the CM2 identified on the MRI head scan. Intraoperatively, a 1.5 × 1 cm pale lesion was incidentally discovered in the fourth ventricle, mimicking the cerebellar tonsils; this lesion was excised under microscopic guidance. An expansile duraplasty was performed to increase CSF flow following the foramen magnum decompression.

Histopathological examination confirmed a CNS WHO Grade 1 choroid plexus papilloma. This lesion was not identified antenatally, due to the small size of the lesion, and it mimicked cerebellar tonsillar herniation that is noted with the myelomeningocele.

### 2.6. Imaging, Syringomyelia and CM2

MRI brain: Corpus callosum was dysplastic and appeared deficient at genu and splenium. Features were in keeping with known CM2—there was also evidence of tectal beaking, a small posterior fossa/low torcula and descent of cerebellar tonsils and cervicomedullary junction. Cervicomedullary junction was at approximately C2/3 level. Cerebellar tonsils are difficult to identify (atrophic at this stage), but at the level of C2.

MRI spine: There were focal dilatations of the central canal from C5‐T2 (AP diameter 1.7 mm) and from T6‐9 (AP diameter 1.5 mm), with visible central canal in the remaining cord suggestive of syringomyelia. At the distal cord/conus (L2/3‐L4), there was a cystic dilatation of the central canal measuring 3.1 mm (AP).

A defect was noted in the posterior elements from the level of L4, inferiorly, with expansion of the dural sac and a low‐lying conus/neural placode. Scan also revealed a bilateral lower‐lobe collapse/consolidation and a small volume intra‐abdominal free fluid (at least related to VP shunt).

### 2.7. Postoperative Imaging

Clinical examination revealed a soft abdomen, with bowel sounds present and no organomegaly. Some apnoeic episodes remained; loud breath sounds and a flow murmur were present, for which an echocardiogram later found normal results.

### 2.8. Patient Respiratory Arrest and Recovery

Despite successful resection of CPP and decompression of CM2 (Figures 4 and 5), in addition to respiratory support, she continued to experience cyanotic episodes. Unfortunately, she experienced a subsequent respiratory arrest necessitating a ventilator and care in the intensive care unit.

**FIGURE 4:**
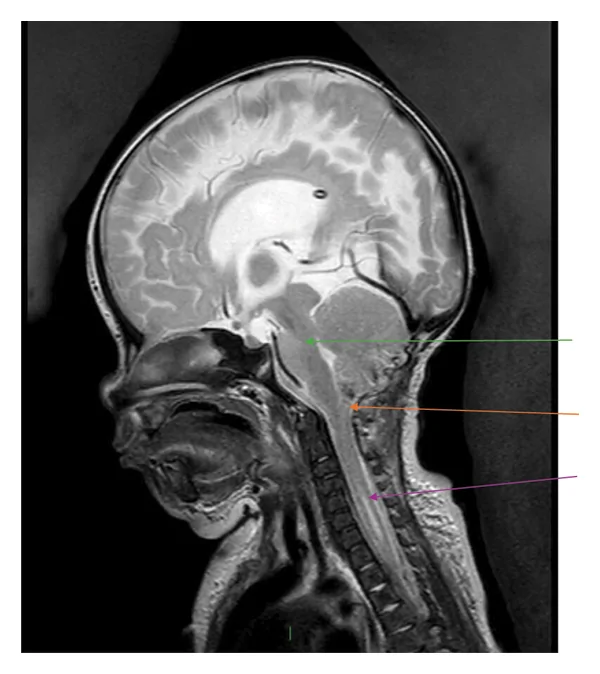
Three‐month postoperative T2‐weighted sagittal MRI without contrast demonstrating resection of the tumour (green arrow) and improvement in cervical syringomyelia (purple arrow) and CM2 (orange arrow).

**FIGURE 5:**
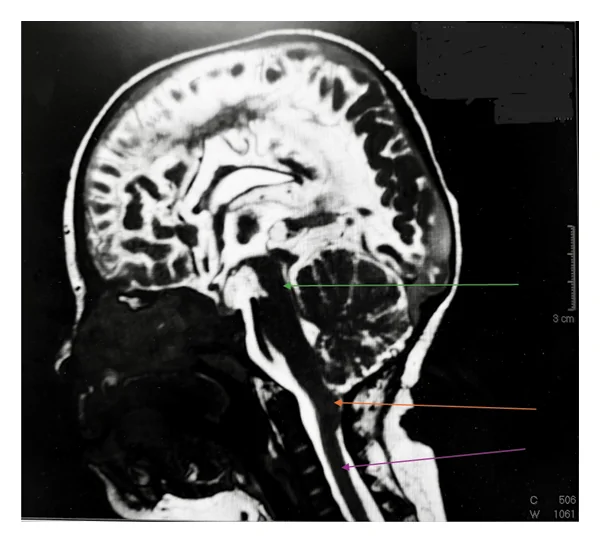
Three‐month postoperative T2‐weighted sagittal MRI demonstrating resection of the tumour (green arrow) and improvement in cervical syringomyelia (purple arrow) and CM2 (orange arrow).

The MDT collaborated with input from ICU, respiratory and neurosurgery, and the patient was slowly weaned from the ventilator and improved from a palliative care status to receiving active treatment and went home on oxygen.

### 2.9. Second Hospital Admission, Urinary Tract Infection and Apnoeic Episodes

Unfortunately, desaturations in the community brought her into hospital once more. On examination, she was tachycardic, with a heart rate of 170 bpm, with a subcostal recession along with loud breath sounds and a flow murmur. She was started on continuous positive airway pressure (CPAP), positive end expiratory pressure (PEEP of 5 cmH_2_O) and FiO_2_ 30%.

Whilst in hospital, she developed a urinary tract infection (UTI), and when this occurred, the apnoeic episodes were observed to increase. Once the UTI was treated, an observed reduction in frequency of apnoeic episodes was noted. At this time, a working diagnosis of autonomic dysreflexia was made, where it was hypothesised that the bladder was being irritated by the infection, which may have caused the apnoeic episodes and disordered breathing. However, no definitive cause of the apnoeic episodes was found, and it is possible that a number of different pathologies contributed to the apnoeic episodes and not one sole cause.

### 2.10. Follow‐Up

The patient is now home in the community with oxygen therapy and BiPAP and has made significant improvement since her ICU admission. She remains under the care of the paediatric and respiratory teams. Further imaging (Figures 5 and 6) has demonstrated a resolution of the syringomyelia.

**FIGURE 6:**
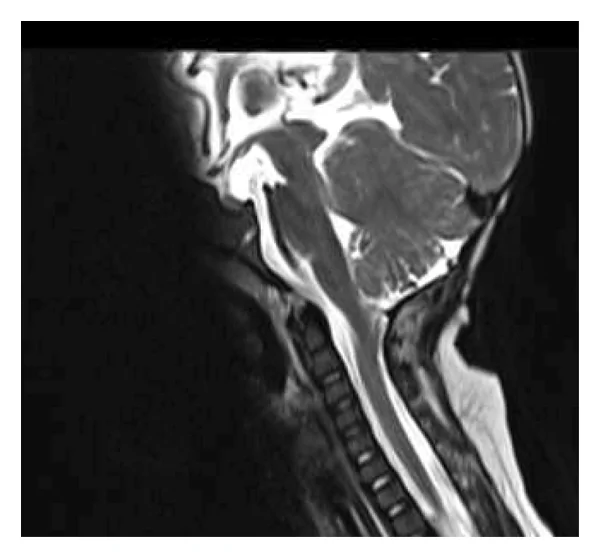
Further imaging one‐year postoperative T2‐weighted sagittal MRI demonstrating complete resolution of cervical syringomyelia.

### 2.11. Histopathology of the CPP

There was debate about the classification of the tumour found incidentally, and whether it was a Grade 1 or Grade 2 CNS WHO CPP. The focal increase in proliferation hinted at the possibility of a Grade 2; however, overall, there was a low proliferation, and it was decided on a Grade 1 classification after MDT discussion with neuropathology.

The tumour specimen (6 × 5.3 mm) had cuboidal shaped tumour cells arranged in a single layer with vascular central core. There was extensive haemorrhage and no evidence of necrosis. Immunohistochemistry demonstrated pancytokeratin and S‐100. There is focal expression of GFAP. ATRX is positive. IDH1, H3K27M, p53 and EMA^∗^ are negative. The proliferation index is predominately low but focal increased.

^∗^Please see the glossary section for explanation of these terms.

## 3. Discussion

This case report provides some insight into the rationale for diagnoses behind the apnoeic episodes in a neonate with multiple pathologies affecting the brainstem and craniocervical junction. Despite extensive discussion between MDT specialities including neurosurgery, respiratory specialities, intensivists and paediatricians, a single aetiology of the apnoeic episodes remains unclear. Multiple aetiologies may be a possibility to consider.

The association between treating the observed UTIs and the improvement in the apnoeic episodes found much later in the patient’s journey creates an avenue of questions surrounding the relationship between possible bladder irritation and changes in breathing.

The number of neurosurgical pathologies which originated around the brainstem also called into question whether pressure, disruption of the respiratory centres in this area and/or surgical intervention for choroid plexus tumour and decompression of CM 2 may have affected the neonate’s breathing.

We have further described some of the literature discussion points, which explore possible reasons for the aetiology(ies) below.

### 3.1. Urinary Infections and Associations With Respiratory Dysfunction

UTIs in infants may be associated with respiratory issues and depression, as research has found that UTIs are present in 10% of children aged 0–24 months who are hospitalised with bronchiolitis [5]. In our case, the temporal association between urinary tract infection and increased apnoeic episodes raised the possibility that bladder irritation or autonomic dysregulation contributed to the respiratory events, leading to the apnoeic episodes described as autonomic respiratory dysreflexia. Treatment of the UTI with appropriate antibiotics did help in the case as there was reduction in the apnoeic episodes, although oxygen supplementation was still required in the community.

### 3.2. Pressure in and Around the Brainstem

The number of different congenital neurosurgical pathologies may have contributed to the presentation.

Research evidence highlighted that symptoms of brainstem dysfunction can include impaired respiratory drive, which are strongly related to myelomeningocele, consistent with this case [3].

CPP may cause direct compression of the brainstem through the wall of the fourth ventricle and cause disordered autonomic control and sleep‐disordered breathing [6]. Both the CPP and CM2 could have exacerbated the hydrocephalus and possibly influenced the level of hindbrain descent through the foramen magnum in Williams’ hypothesis [7].

Another study has described CM2 displacing the fourth ventricle, altering CSF flow [8], and has suggested that CPP could be a reactive lesion related to chronic compression or ischaemia due to CM2 [8]. Arachnoid cysts can be found at the craniocervical junction [9], and interestingly, craniocervical decompression for CM2 can result in ventrally positioned arachnoid cysts [10], indicating a possible mechanism of abnormal cellular growth triggered by structural changes. The idea that structural change may be associated with the development of pathology is perhaps evident in a case where a 22‐year‐old female presented with a CPP in the posterior third ventricle, 7 years post Chiari malformation (CM) decompression. Research demonstrates that there may be instances where CPP pathology may develop and found in association with CM [11, 12].

In a cohort of adult patients, CM I was associated with sleep apnoea pre‐ and posterior decompression [13]. The apnoea was seen to improve after the decompression but not completely resolve, with 4–6 out of 8 participants still reporting sleep apnoeic symptoms postoperatively. Although it is important to note the distinction between adult patients and paediatric, there may be some overlap between the apnoeic symptoms and possible compressive pathology around the brainstem [14].

Another mechanism of acquired Chiari I malformation may be secondary to an over‐draining VPS, which can cause herniation of the cerebellar tonsils in response to the pressure changes. Although this is an important differential to consider, in this case, we have a background of Chiari II pathology and evidence of a ventriculoperitoneal shunt which was not over‐draining.

The autonomic dysfunction of respiratory compromise may have risen from persistent pressure changes around the craniocervical junction [15] and compression of the brainstem [13] by the combination of pathologies and may cause a sudden loss of spontaneous respiratory output [16]. Interestingly, autonomic nervous system dysregulation was also seen to play a role in other pathologies such as chronic obstructive pulmonary disease, mechanistically through, cholinergic hyperactivity driving airway constriction [17].

### 3.3. Hydrocephalus, Brainstem Herniation and Their Relationship With Apnoea

The prevalence of sleep apnoea in different types of hydrocephalus has not been widely studied, except in those with CMs. In patients who have a CM, the hydrocephalus severity has been associated with worsening severity of apnoea, which demonstrates a positive correlation between the two. However, the specific pressure on the respiratory centres from a hydrocephalus needs further investigation for definitive conclusions.

Interestingly, there is also a high prevalence of patients with sleep‐disordered breathing, which may also include apnoea in idiopathic normal pressure hydrocephalus. Transient elevations of PaCO_2_ have been found alongside hydrocephalus which reflects that CO_2_ is an essential and primary factor in altering intracranial pressure, which changes in individuals with hydrocephalus (13).

Williams theorises that lower spinal pressures relative to intracranial pressure in hydrocephalus create a gradient that displaces the hindbrain through the foramen magnum [5]. Another way in which hydrocephalus may be related to CM is thought to result from posterior fossa hypoplasia in CM2, and improving foetal CSF flow could reduce the impact of CMs on infants (3). Both of these may highlight other mechanisms where hydrocephalus may associate with CM and again sleep apnoeas. Interestingly, the researchers proposed that hindbrain herniation, myelomeningocele level and whether a VPS was needed did not predict the number of apnoeic episodes.

### 3.4. Learning Points

A learning point from this rare case is that the clinical team could consider nonsurgical compressive causes, such as a UTI, in the differential diagnosis for respiratory depression in young infants, even if they have undergone neurosurgical procedures affecting the brainstem.

## 4. Conclusion

Although the urinary tract infection was temporally associated with an increase in apnoeic episodes, a diagnosis of autonomic dysreflexia remained speculative and was not formally established. The temporal improvement after treatment of the urinary tract infection raises the possibility of bladder‐mediated autonomic or respiratory dysregulation, but this requires further evaluation. The absence of complete postoperative improvement does not exclude a contribution from brainstem compression or dysfunction. There is a lack of evidence to suggest that other contributions, such as CM2 and syringomyelia, were or were not involved due to a lack of systemic evaluation. The autonomic dysreflexia was unsupported by the literature, and the proposed association with UTIs was not established. Further investigation is needed to ascertain the causes of apnoeic episodes in infants with multiple neurosurgical pathologies around the brainstem.

**Nomenclature**
Pancytokeratin: A broad‐spectrum marker used in medical diagnostics, used to identify epithelial cells in tissue samples
S‐100: A protein marker, present on the surface of the tumour, used for identification of tumour type
GFAP: Glial fibrillary acidic protein, an intermediate filament protein expressed in astrocytes
ATRX: A mutation in various tumours, including neuroblastoma and neuroendocrine, which disrupt the ATRX protein and lead to chromosomal instability
IDH1: Isocitrate dehydrogenase 1 is an essential metabolic agent in the citric acid cycle. Changes in its expression are linked with mutation in different tumour types
p53: A tumour suppressor protein and biomarker which detects DNA damage. A positive result indicates neoplastic change
EMA: Epithelial membrane antigen is a transmembrane protein, which is used as a diagnostic tool. Some types of tumours consistently express EMA particularly meningiomas and some breast cancers
Myelomeningocele: This is the most severe kind of spina bifida, where the layer of the neural tube does not completely close around the spinal cord in utero. It is associated with Chiari malformation, syringomyelia and hydrocephalus
Syringomyelia: A fluid‐filled cyst within the spinal cord
Chiari II malformation: It is, also known as Arnold Chiari malformation, a congenital condition, associated with spina bifida, where the brainstem and cerebellum extend into the spinal cord

## Author Contributions

Harris Shahid: writing–original draft, writing–review and editing, investigation and research. Priya Kadam: conceptualisation, visualisation, methodology, investigation, writing–original draft and writing–review and editing. Chandrasekaran Kaliaperumal: conceptualisation, methodology, resources, writing–review and editing and supervision.

## Funding

There are no funding sources to declare.

## Ethics Statement

Institutional Review Board approval was not required for this single anonymised case report; this has been added to the manuscript.

## Consent

Informed consent was obtained from legal guardian to publish this case report. Images were anonymised, and that the necessity of an ethics committee was evaluated according to institutional policy.

Written informed consent has been obtained from the study participant’s parents to participate and to publish.

## Conflicts of Interest

The authors declare no conflicts of interest.

## Data Availability

All data used in this report are from the patient’s hospital notes and, therefore, are not publicly available. Further enquires can be directed to the corresponding author.
